# The Veterinary Pharmacopœia

**Published:** 1818

**Authors:** James Carver

**Affiliations:** Professor of Animal Medicine


### VETERINARY PHARMACOPŒIA,

OR,

### INSTRUCTIONS

FOR

### Compounding Horse Medicines,

AND

PREPARING THE VARIOUS SUBSTANCES

*EMPLOYED IN VETERINARY PRACTICE,*

AT THE DIFFERENT

Veterinary Colleges of Europe and British India:

WITH

A LARGE COLLECTION OF VALUABLE

### RECEIPTS,

OF

ESTABLISHED EFFICACY;

Tried on Condemned Animals at those Institutions.

*BY DR. J. CARVER, P. A. M.*

### PREFACE.

It is but within these last thirty years, that the Veterinary art has assumed the form of a science, and thereby acquired a distinct appellation on a solid foundation. Receipts handed down by traditionary skill, in which ingredients were accumulated without judgment or discrimination, constituted the principles and practice of what was termed Farriery; a name which it derived from the occupation of those persons who practised it, and who, in general, were smiths or workers in iron (Ferracius).

To attempt to distinguish the causes of the Horses' diseases, was far beyond their little skill; and, in general, random trials of a few burning medicines formed their boasted practice.

The science, however, at one time, began to rise above the order of smiths, and attracted the notice of medical practitioners; but it was not thereby greatly improved, as those physicians of a day were not aware of the difference, *which has since been found* to exist, between the structure and economy of the horse, and that of the human subject; nor had they any idea, that this dissimilarity required much consideration with respect to diseases, and the effect of medicine on the quadrupeds; and one thing which never would have escaped their notice, was the horizontal position of the horse, as contrasted with the upright form of man. This publication will, however, enable every person to form a proper judgment betwixt the constitution of a horse, and that of man; also, the different effects of the same medicines in both. Thus, *Arsenic* has been given to a horse to the quantity of some drachms while one eighth of a grain is the proper dose for the human subject. *Tartar Emetic*, also, a medicine equally active, is now, in some cases, administered to the amount of even some ounces daily; for the human subject, a single grain is a dose, and often too large a one. *Blue Vitriol*, has also been given in the same manner, to the amount of some drachms a day; *Verdigris* also, to the same extent; as *Henbane, Hemlock, &c. &c. &c.* Corrosive Sublimate, also, has been given under my own inspection, when in charge of the Hospital stables at the Royal Veterinary College, to *condemned horses*, without producing any possible effect. On this subject I might enlarge; but I hope sufficient has been said, to show what is due to the improvement in this department of anatomy and physiology, as well as medicine; and to none am I more indebted, than to my worthy instructors: professor Coleman, William Sewell, esq. assistant professor, and Bracy Clark, and others of the medical profession; for their correct anatomical views of various parts of the animal; for their tracing the cause of many diseases, as well as the difference of structure in both, to their source, on which the most erroneous ideas have been entertained; and for explaining their opinions on a proper analogy, between the maladies of animals and man; thus rendering their explanation easier, and better understood.

It is only since the establishment of Veterinary institutions in all parts of the world, except America, that the anatomy and physiology of the horse, and other quadrupeds, have been properly investigated; and the effect of medicines on their bodies correctly ascertained, by numerous and appropriate experiments on condemned animals, both in health and disease, so that a secure foundation is now laid; and I hope, the time is not far distant, when we shall derive the same satisfaction in seeing the same progress of improvement in this country.

And, though many books have been copied in this country, from books of Farriery, concerning the diseases of horses and ttle, those people who put them forth knew not, that the

Veterinary Art had assumed the form of a science, and that they were only catch-penny cow doctor books, that could contain very little or no scientific information concerning the diseases of quadrupeds; but more particularly, the therapeutical parts, which relates to those medicines proper for their diseases. Such a work as this, therefore, appeared to me, a desideratum in the Veterinary Art; and has induced me to add to it, the College Pharmacopœia. This will comprehend the materia medica, comprising directions for forming and compounding the various compositions, in the most convenient and efficacious manner; together with many valuable receipts and discoveries, by the Brahmins and Veterinary physicians of the college of Bencras, in India; the whole forming a complete system of *Therapeutics, instructing the inexperienced* how to distinguish the purest and most genuine drugs, and to compound them in such a way as will enable him to *combat*, with success, the various diseases to which horses are liable.

Having commenced this useful task with the sole desire of making myself useful, by the instruction I shall convey in these productions, I feel it also a duty incumbent on me, to extend my labours by lectures; and to present a descriptive catalogue of such medicines only, as my own experience enables me with practical confidence to recommend to the practitioner's notice—it being my firm intention, in these publications, to teach the practice of the art on principles, and not receipts only; nevertheless, they will be a safer guide to those not medically educated.

In doing this, I cannot do better than to give, and recommend the same advice as imparted by one of our first and most experienced professors in Europe; and, in fact, to convey every species of information that may be useful to the junior practitioner. In the Pharmacopœia which is intended to be added to this work, instructions will be given for compounding and mixing medicines in such a way, that they may mutually assist each other in their curative operation, and sometimes produce effects that cannot be obtained from either of them individually; for many persons undertake to compound veterinary medicine who are unacquainted with chemistry, and not aware that, by improper mixtures, the original qualities of the ingredients may be destroyed; thus, by mixing many of the acids with simple soda, (many of which are powerful caustics,) an innocent salt only is obtained; and many, very many, are the mistakes of this kind, to be found in almost every book of Farriery which we find written by those, who, *not emerging* from a school of proper instruction, cannot of course be relied on; for how frequently do we find them recommend, and direct heterogenous mixtures, uniting medicines of opposite qualities in the same ball or drench.

In this appendix, I have, however, endeavoured to avoid those errors; and no medicine will be recommended in this Pharmacopœia, but such as have undergone the test of experiment, either in the London or other Veterinary Colleges of Europe and British India.

J. CARVER,
*Professor of Animal Medicine.*

### VETERINARY PHARMACOPŒIA.

### ABSORBENTS.

The efficacy of this class of remedies is supposed to consist in their tendency to correct diseased acidity in the stomach. Horses are sometimes disposed to eat their litter in preference to hay; and not unfrequently, they have a propensity, (but more particularly in the hot climates of India,) to swallow earth or any kind of rubbish. The horses of that country being more prone to this act than those of other climates, I account for, by the earth of that country being always more or less impregnated with salt: it is also supposed to arise from the irritation of an acid in the stomach; but as this organ in the horse has but a small portion of secreting surface, so is he less liable to affections of this nature than any other animal.

In horned cattle, complaints, apparently originating from this source, are more common. Horses, cows, calves, and sheep, are sometimes benefited by chalk; which, mixed with chaff, or cut hay, is found very useful, when acidity of the stomach arises from debility of that organ. It is, therefore, recommended as the best anti-acid hitherto discovered in veterinary practice.

No. I.

| | |
|---|---|
| Prepared chalk . . . | 3 drachms |
| Catechu . . . . | 3 drachms |
| Aromatic powder . . . | 2 drachms. |

No. II.

| | |
|---|---|
| Pulverized ginger . . | 2½ drachms |
| Catechu and quassia, each . | 2 drachms |
| Prepared kali . . . | 3 or 4 scruples |

Oil of carraway, oil of peppermint, oil of chamomile, oil of thyme, or any of the essential oils, 12 or 15 drops.

No. III.

Catechu, columba root, soda, or prepared natron and cassia, well pulverized, each 2 drachms.—*Mix.*

Note.—These medicines are to be made into balls, with a little linseed, carraway seed, or any kind of flour, with syrup, honey, or molasses.—One to be given every morning.

### ACIDS,

In chemical language, are a class of salts; but among practitioners, they familiarly express whatever produces the sense of sour to the taste. They are obtained from the three kingdoms, the *mineral*, the *animal*, and *vegetable;* and such as are in use in veterinary practice, are described under proper names in the materia medica.

### ALOES,

Is the inspissated juice of certain plants, and is a very important article in the list of Veterinary medicine. Aloes form the most effectual purge for the horse we are now acquainted with; too much care, therefore, cannot be taken, to procure them genuine. The different sorts of aloes are distinguished by their names, and the plants from which they are procured.

Every practitioner, however, should purchase them by the quantity, and have them powdered under his own eye, as being the surest way to prevent adulteration.

Aloes are of several sorts, though the three most in use are, the *Barbadoes*, the *Socotrine*, and the *Cape*. The *Socotrine* was formerly the most used, though I have often been disappointed in their effects; having, however, given a fair trial to those above, I have uniformly found the Barbadoes the best, and the most sure, certain, and active in their operation. They are, for various reasons too lengthy for insertion in this place, to be preferred. Suffice it, therefore, to say, that they are less brittle, less fragrant, and less bitter, I think, than the others, though some druggists say they are more intensely bitter: these cannot be well adulterated without detection, they being of a darker colour than any of the others. It should, however, be understood, that if given too largely, and the horse treated improperly, they must prove fatal; as for instance, when you have given the strictest orders (in winter) to give the horse moderately warm water, and have gone and found the servant leading the poor animal to the pump, surrounded several feet thick with ice;—and all this *with the severest imprecations* against the doctor, because he directs John, Tom, Dick, or Harry, to take the trouble to warm a tea kettle of water over his master's kitchen fire. This neglect often dooms many a poor animal to his long home; for by the administration of cold water, during the action of the physic in the intestines, it is prevented from passing through in the proper given time; and the consequences are sometimes serious, and often fatal.—"The doctor has killed my master's horse," says the groom; "but it was because I did not obey the doctor's instructions."

Aloes is very often given, or administered, under various forms, and in various ways besides that of physic. As an alterative, as a stomachic, as a vermifuge, as a cataplasm, and as an external detergent and stimulant application; it is also used as a tincture, &c. As a cataplasm, it is very much used among the natives of the east; in this form, and as a plaster, as well as a poultice, I have been eye witness to its powerful effect on the elephant, the camel, the horse, and other quadrupeds—which shall be enumerated under their proper heads alphabetically. In winter time, they do not powder readily, and if laid by even in the powdered form they will unite into a solid mass again; they should, therefore, be mixed and formed into balls, with half their weight of any kind of oil; in this manner, they will keep of a uniform small and proper consistence, and never harden; and, of course, readily dissolve in the stomach without griping. If, however, an occasion should happen when they are in your possession in the solid form, you may readily dissolve them in lard or molasses, over a gentle heat; in this way, the ball may become too large to be taken at once, in which case they must be given at different times. A tincture or watery solution should always be kept by every person in the habit of using them frequently, in large establishments; in this form aloes is very useful, on account of its quicker action. I generally make it in the same way as prepared at the different colleges, by pulverizing a pound of the mass, and infusing it in a warm place (in the summer, in the sun—in the winter, by the fireside) in a pint or a pint and a half of best proof spirits, for three, four, or even five days; after which you may add soft water that has been boiled, about two quarts, and then bottle for use; when necessary to be given, shake your bottle, and give two, three, four, or even five ounces, as circumstances may require. It should be remembered that the large doses are only required in cases where the peristaltic motion of the intestines are supposed to be lost: as in Tetanus, or Lock Jaw, Staggers, &c.

No. I.—*Very mild.*

| | |
|---|---|
| Barbadoes aloes . . . | 6 drachms |
| Pul. cinnamon . . . . | 1 drachm |
| Ol. aniseed . . . . | 20 drops |

Peppermint water sufficient for a ball.

No. II.—*Mild.*

| | |
|---|---|
| Socotrine aloes . . . | 1 ounce |
| Ginger . . . . . . | ½ drachm |
| Ol. thyme . . . . . | 40 drops |

Peppermint water sufficient for a ball.

No. III.—*Strong.*

| | |
|---|---|
| Barbadoes aloes . . | 8 drachms |
| Ginger . . . . . . | 2 drachms |
| Volatile oil . . . . | 40 drops |

Peppermint water sufficient for a ball.

No. IV.—*For carriage or cart Horse.*

| | |
|---|---|
| Barbadoes aloes . . . | 8 drachms |
| Calomel . . . . . . | 2 drachms |

Peppermint water and linseed, sufficient for a ball.

NOTE.—It should be observed, that when physic is given to horses at grass, one-fourth of the aloes should be subtracted.

### ALTERATIVES,

Are medicines that act both gradually and medicinally on the constitution, in a slow and almost imperceptible manner. The usual Alteratives used among Farriers, are, *Antimony*, Nitre, Sulphur, *Spices* and *Resin*, which are by them usually given together; but a better acquaintance with the art has taught us better, and we now sometimes add several of the Gums—*Fox Glove*, *Arsenic*, *Mercury*, and some of the *wood barks*, *&c.* A change in diet will also, sometimes, act as a powerful alterative. Nitre, in drops, from two to six drachms, increases the urinary discharge, and keeps down the accumulation of fluids in swelled heels, and other œdematous enlargements. *Antimony*, is given in several forms, from half a drachm to six, for affections of the skin, hide-bound, &c. Antimony, is an excellent alterative, in doses of one or two drachms. *Antimonial Powder*, and *Tartar Emetic*, are both prepared of antimony, which is certainly one of the best medicines we have in use. *Cream of Tartar*, is also an excellent alterative, when combined with mercurials and sulphur, for surfeits, &c. &c. *Resin*, is an active diuretic, and should be classed among the *Diuretic Alteratives*, such as Nitre, Soap, Turpentine, &c. These diuretic alteratives are used as a preventive when a horse is subject to swelled legs.

### THE DIAPHORETIC ALTERATIVES,

Are considered, when applied in affections of the skin, as intended to increase insensible perspiration; they also give a gloss to the coat. The complaints in which this class of alteratives are used, are such as surfeit, hidebound, and insensible perspiration; they are also given to remove an undue determination of blood to any internal organ, or to diminish general plethora. They, however, seldom prove effectual, unless assisted by good grooms, exercise, &c.

### TONIC ALTERATIVES,

Such as Arsenic, is considered as one of the most powerful kind in the horse; it must nevertheless be used with caution, and should not be administered but by the hand of a veterinarian, who alone can know how to correct its effects, if improperly given in too large a dose, or on an empty stomach. *Tonic Alteratives*, are also composed of *Copper*, *Zinc*, *Iron;* there are also Vegetable Tonics, such as the different barks, gentian, quassia, snake root, and other bitter roots; all of which will be noticed in their proper places. The alteratives of farriers in general, are not composed with any distinction; laxatives, diuretics, &c. being all mixed without discrimination. They generally mix sulphur, nitre, resin, antimony, and sometimes turpentine, with an intention to remove diseases supposed to arise from obstruction; this mechanical mode, however, of removing obstructions, is now totally disregarded, as incompatible with our present knowledge of physiology.

### THE MERCURIAL ALTERATIVES,

Are composed of calomel, and corrosive sublimate; they are useful in all herpetic affections; and as a vermifuge, in doses of a scruple to a drachm, and a drachm and an half, does wonders. They should not, however, be continued beyond the third dose, or salivation may be produced. In cases of Farcy, Glanders, and Grease, corrosive sublimate may, in doses of ten and twelve grains to a scruple, be given with the same precaution, as it may produce inflammation of the stomach. It should also here be noticed, that arsenic is not given with the same intention as the last articles, but as a tonic in *protracted debility;* it is, in small doses of one, two, and three scruples, very useful; and in all watery accumulations, fox glove is a powerful alterative, given in the same quantities.

### LAXATIVE ALTERATIVES,

Are very often substituted for purgatives, with great advantage. For horses that are troubled with worms, when too weak to take the stronger purgative which is employed to destroy worms, calomel, in small doses of one, two, or three scruples, I have found very serviceable. I have also given only common salt, to the extent of two, three, and five ounces, which sometimes, in a few days, opens the bowels, and proves very serviceable; but people living near the sea shore, should give the sea water in preference. In obstinate cases of Grease, and in Chronic Inflammation of the Eyes, sea water is also serviceable. But the strongest laxative alterative is aloes, given in a drachm, a drachm and an half, or two drachms, with an equal quantity of soap; and sometimes we add ten, fifteen, or twenty grains of calomel.

### *A Laxative Alterative.*

| | |
|---|---|
| Barbadoes aloes . . . | 10 drachms |
| Castile soap . . . . | 1 ounce |
| Ol. cloves . . . . . | 20 drops |

Aniseed powder and syrup sufficient to make four balls, to be given every night.

### *A Diuretic Alterative.*

| | |
|---|---|
| Nitre . . . . . . | ½ an ounce |
| Sulphur and antimony, each . | ½ an ounce. |

Linseed sufficient to make the ball with any kind of syrup.

### *Diaphoretic Alterative.*

| | |
|---|---|
| Emetic tartar . . . . | 3 drachms |
| Sulphur . . . . . . | 3 drachms |
| Nitre . . . . . . | ½ an ounce |

Linseed and honey sufficient to make two balls, one to be given, for a week, every night in feed.

### OF THE ACIDS.

In veterinary medicine they come under a variety of heads, and are all used in their turn; as the *Sulphuric, or oil of Vitriol,* the *Muriatic,* the *Tartareous,* the *Nitrous,* and the *Acetous Acids.*

### ÆGYPTIACUMS,

Are of various kinds, and when properly mixed, and properly applied, are, in all foot cases, worthy of more attention than perhaps one half the compositions in the materia medica. They are generally mixtures of *Verdigris, Honey, Vinegar, Alum, White Vitriol,* and *Borax.* This invaluable medicine is also used in Grease, Cracks, Thrushes, and particularly Canker of the feet, Ulcers, &c.

### *Ægyptiacum Ointments.*

Take of pulverized Verdigris, in powder, 6 ounces; Honey, 20; Vinegar, about 7, 8, or 9 ounces; which must be boiled over a gentle fire to the consistence of an ointment.

OR,

Take of Honey, 1, 2, or 3lbs.; Blue Vitriol, Verdigris, and burnt Alum, in powder, each 1½ ounce; boil them over a gentle fire, till they have acquired a proper consistence, and a reddish colour.

### *Another preparation of Ægyptiacum, well adapted for Running Thrushes and Canker feet.*

Take of burnt Alum, blue Vitriol, and Verdigris, each 2 ounces; Corrosive Sublimate, 1 drachm, in powder; Vinegar, about 6 ounces; Honey, about a pound or pound and a half; sufficient to make it of a proper consistençe, when boiled over a gentle fire.

### ÆTHIOPS MINERAL.

*(Sulphuretum hydrargyri ingrum.)*

This medicine, on account of its very high price, is but very little used in horse practice. Its proper virtues, also, I believe, are not sufficiently known; it was formerly much used among the ancients, and the moderns have again brought it into use, although very expensive. In Surfeits, and foulness of the skin, it has performed wonders, and may with safety be used to the extent of five or six drachms, mixed with ten or twelve of cream of tartar, daily. It is now considered as one of our best alteratives.

### ETHER,

Has also proved itself a very valuable medicine; but is too expensive to be brought into general use as a veterinary medicine.

Nitrous Ether, or the sweet spirits of nitre, is a good and valuable medicine, and will no doubt come into more general use as it becomes better known. A drachm of Sulphuric Ether, mixed with eight or ten of water, is now found to be an excellent wash or salve in the last stages of ophthalmia.

### ALUM,

*(Alumen.)*

Is a saline compound body, composed of sulphuric, vitriolic, alumine or pure clay. It is used in diarrhœa and diabetes as an internal astringent. Externally it is used in many forms. I have used it with great success on ulcers to destroy fungus, commonly by old women called proud flesh, and it may not be inapplicable in this place to state, that what they sometimes term proud flesh, is the healthy granulations of a wound, which they keep discharging, under the supposed but improper idea of its being proud flesh; consequently they do much mischief by keeping open for weeks a healthy wound, which might, if let alone, be healed in a few days.

It is an excellent styptic to stop bleeding, and if sprinkled on the surface of the orifice will constringe and plug up the mouth of the bleeding vessel: it is also a useful astringent in inflammations of the eye, and the whey of this drug makes an excellent eye-wash, and for horned cattle in fluxes of all kinds, is one of the best drinks I know of. Vinegar and molasses, mixed with water, as a common drink in those cases attended with fever, I have also found of great advantage. It also makes a good astringent clyster, both for the horse and horned cattle, but particularly for calves.

NOTE.—Alum for external purposes is also a very useful remedy for Grease, when finely pulverized and sprinkled on the diseased parts. *Burnt alum* is made by putting any quantity of the crude alum in a pot or iron ladle, and keeping it over a gentle fire until all the watery particles are absorbed or evaporated. It may then be easily converted into a pulverized substance. But if exposed to too strong a heat, it becomes decomposed, and of course useless.

### AMMONIA.

*(Ammonia carbonas.)*

Ammonia is now made and used under various forms, but according to the modern term is now called volatile alkali, and is principally composed of bones, or sal ammoniac. It is said to be an excellent stimulant in fevers; it is also an excellent ingredient in all liniments, and when mixed with oil and camphor, which has been dissolved in spirits of turpentine, joined with the white of one egg, forms an excellent application for swellings, strains and bruises.

The prepared Ammonia, given in the latter stages of fever, in drops of one or two drachms, under approaching debility, will be found a serviceable medicine, and if mixed with the compound spirits of ammonia, and the fetid spirit wherein asafœtida has been mixed, its qualities will be greatly improved. Its virtues, given in this form, has proved it to be an excellent antispasmodic in nervous complaints. In veterinary practice it has not until lately been much used with us, though among the Persians and veterinarians of the East, the fetid spirit of ammonia has been long used and known, and when squills are added to the fetid spirit, it will be found an excellent remedy in cough and catarrh.

### THE SPIRITS AND SALTS OF HARTSHORN,

Are nearly the same as the prepared, but being distilled from the bones and horns of stags, it is highly impregnated with animal oil, which gives it that peculiar fungous smell and increases its antispasmodic qualities.

### ANISE SEED,

Has been long in use among the old farriers of the day, and if they can be procured genuine, when powdered, may with great propriety be used with other warm aromatics, or as a help in mixing up balls.

### ANODYNES,

In the human subject, are medicines that quiet pain and produce sleep; but as a veterinary medicine, none of our present professors are of opinion, that either opium or any other medicine yet discovered is capable of producing this effect on the horse. The stomach of the horse being but small, and being also divided into two portions, and having but little secreting surface, acts, like anodynes, by a sympathetic effect; so in him this class is not numerous, and opium, which is the grand anodyne of the human subject, is also the sheet anchor of the veterinarian; yet we have found in veterinary practice that camphor and ether will, when properly applied, mitigate pain. Opium, when administered to the horse, is generally given in doses of from 1 to 3 drachms; but when the pain is produced from inflammation it should not be employed.

In symptoms of inflamed intestines, or inflamed bowels, opium would do much injury, but in flatulent spasmodic colic, commonly called gripes, it seldom fails of giving relief.

Some veterinarians belonging to some of the colleges on the continent, have, I believe, attempted to prove that this invaluable antispasmodic is nearly inert in its effects on the horse; but it should at the same time be taken into consideration, that no theory can possibly overturn facts, which have, and must on further trial incontrovertibly prove of public benefit. Opium has, on a fair trial, proved itself to have the most salutary and active effect on the horse; in spasmodic diseases its effect and benefits have been particularly observable, in the doses here prescribed; in clysters also its beneficial effects have been particularly observed; it has also been very beneficially employed in assisting the action of astringents, in diarrhœa and profuse staling, and when united with alum and catechu, it is singularly efficacious. When given in doses of one and two drachms every six or eight hours, it increases the pulse, and proves a useful auxiliary in debilitated stages of fever.

*Anodynes.*—No. I.

| | |
|---|---|
| Opium . . . . | 1½ drachm |
| Best brandy . . . . | ½ pint |
| Camphor and soot . . | 2 drachms |

No. II.

| | |
|---|---|
| Opium and camphor, each . | 2 drachms |
| Brandy . . . . . | ½ pint |
| Peppermint . . . | 4 ounces |

No. III.

| | |
|---|---|
| Liquid laudanum . . | 4 ounces |
| Brandy . . . . . | 1½ pint |
| Camphor (mixed in a qt of gruel) | 4 drachms |

NOTE.—To either of these 4 or 5 ounces of *Ether* may be added; but when this last ingredient forms a part, it must be given with great expedition.

### ANTISPASMODICS.

The antispasmodic and the anodyne being nearly the same may as well be classed together. Though the horse is not so subject to spasmodic disease as the human subject, yet as opium, camphor and ether, are the chief ingredients, we will insert them in this place.

No. I.—*Antispasmodic mixture for Locked Jaw.*

Take Opium and camphor, each 2 drachms, Ginger 4 drachms, mixed with syrup for a ball, or with some stimulating fluid, as brandy, about 8 ounces, and given as a drench.

No. II.

Take compound tincture of Cardamoms from 3 to 5 ounces, Peppermint 3 ounces. Then add 1 ounce of Ether.

Lock Jaw is a disease that generally proves fatal to horses; but when the jaws are not firmly set, it may be worth while to try the above medicines. I have known several cases in this city, in which brandy and opium, and brandy and laudanum succeeded. It is however necessary to repeat it, and that in very large and powerful doses (a pint of laudanum to a quart of the best proof spirits, mixed with about a quart of gruel, and given in two drinks) firing, and a very strong blister run over the jaws and all down the spine to the anus, in cases requiring it. This treatment I have seen attended with good effects, and the horse finally saved. The lines of fire should be run across the vertebræ of the neck; one line down and upon the back bone; and two or three lines about two inches apart on each side of it.

The blister for this purpose should be made as strong as the ingredients can make it, and well rubbed in.

No. I.—*Antispasmodic mixture for Flatulent Colic.*

Oil of turpentine 4 ounces; Gruel a pint; add Camphor one drachm and half: and mix for one dose.

No. II.

| | |
|---|---|
| Camphor . . . | 1½ drachm |
| Ether . . . . | 3 drachms |
| Peppermint . . | 2 drachms |
| Oil of turpentine . . | 2 drachms |

Remark.—These are formidable doses, and to an inexperienced person might give alarm. If, however, the symptoms are correct when administered, they will never do harm.

The symptoms of this disease being so nearly allied to inflammation of the bowels, intestines, bladder, &c, they are unfortunately too often mistaken, and the inexperienced practitioner oftener kills than cures. It should therefore be remembered, that this medicine must never be given but when the animal attempts to roll on his back and endeavours to keep in that position. It should also be observed, that when the horse does this, it is a symptom of colic or gripes, in which case you may not only give these doses with the greatest confidence, but if no relief is obtained in two hours, repeat it. I have frequently given it in much larger doses. Should you, however, mistake the symptoms, and give the above when tho animal lays down and paws, and continues to paw *without rolling on his back*, you kill to a certainty. All you have to do is carefully to distinguish the flatulent or spasmodic colic from the inflammatory, and also that which arises from costiveness. Clysters and back-raking tend greatly to facilitate the cure, and prevent its return.

No gentleman who travels much with horses should go without this Instructor, and should his horse be attacked in a remote situation, where no remedy is at hand, how easily a few opium and camphor balls (which he should always carry with him) may be dissolved in a quart of good strong beer and given as a drench.

No. I.—*Antispasmodic mixture for Suppression of Urine.*

| | |
|---|---|
| Purified nitre . . . | 1 ounce |
| Camphor . . . . | 2 drachms |

No. II.

| | |
|---|---|
| Epsom salt . . . | 4 drachms |
| Juniper . . . . | 1 drachm |
| Sapo . . . . | 4 drachms |

NOTE.—These may be mixed and made into a ball with the mucilage of Gum Arabic, or a little Linseed infusion, and given as a drench. I have uniformly used this medicine with success, though I have sometimes found a clyster a necessary assistant. Should the complaint return, give a laxative drench, and repeat either of the above medicines.

It has been the opinion of some of the Colleges that the disease called Staggers is generally occasioned by a diseased state of the stomach. This there can be no doubt of, nor that antispasmodics, judiciously applied, sometimes prove the best remedies.

I have had so much practice in this disease, that I am persuaded copious and timely bleeding is the sheet anchor on which we must depend. I shall, however, give a few receipts, which may perhaps occasionally prove serviceable.

No. I.

| | |
|---|---|
| Ol. Peppermint . . . | 1 scruple |
| Tincture of valerian . . | 1 ounce |
| Asafœtida . . . | 5 drachms—*Mix: 1 dose.* |

No. II.

| | |
|---|---|
| Ether . . . . | ½ ounce |
| Camphor . . . . . | 1 drachm |
| Fetid spirits of ammonia . | 1 ounce |
| Ol. Peppermint . . . | 15 drops—*Mix:* 1 *dose.* |

No. III.

| | |
|---|---|
| Camphor . . . | 1 drachm |
| Salt of hartshorn . . | 2 drachms |
| Asafœtida . . . | 4 drachms |
| Ol. Thyme . . . | 15 drops—*Mix:* 1 *dose.* |

Note.—In Staggers the peristaltic motion of the bowels is rather torpid, and we sometimes cannot get even the strongest cathartics to operate. I would therefore recommend the following purgative:

| | |
|---|---|
| Barbadoes aloes . . . | 8 drachms |
| Calomel . . . . . | ½ drachm |
| Soap, Q. S. for ball | |

No. I.—*Antispasmodic mixture for Cough.*

| | |
|---|---|
| Powdered squills . . . | 1 drachm |
| Camphor . . . . | 1 drachm |
| Opium . . . . . | 1½ drachm |

To be made into a ball with honey, molasses or soap, for one dose.

No. II.

| | |
|---|---|
| Squills . . . . | 1½ drachm |
| Opium . . . . | 2 drachms |
| Balsam of Tolu . . . | 1½ drachm |
| Asafœtida . . . . . | 1 ounce |
| Balsam of sulphur Q. S. for one dose. | |

NOTE.—These balls should be given every night or morning, until you observe the animal gets relief. I have in my practice found them very efficacious, and oftener better than the laxative alterative. Coughs which arise from inflammation or irritation of the throat, constantly harass the animal, and may easily be distinguished; in which case, emollient drinks of linseed and honey may be given as often as the animal feels disposed to take them. This is also an excellent drink for cows and oxen, under the circumstances I have just noticed, though I cannot tell why (unless it is on account of the acid) that horned cattle always prefer the molasses to the honey.

### ARSENIC.

*(Arsenici oxydum.)*

This very powerful medicament has but very lately been properly estimated. The modern veterinarian has at last found it one of the most powerful and excellent tonics, and in doses from ten to thirty grains a day, finely powdered, gives a powerful tone to the stomach of the horse. It however in some cases remains in the constitution, and produces its noxious effects suddenly and irresistibly; it is, therefore, prudent to exhibit it with great caution, and should never, as before observed, be given but by the hand of a veterinarian.

### ANTHELMINTICS.

Of the anthelmintic medicines a great variety of the vegetable class have been supposed to possess the quality of expelling worms; but I have not found any of them effectual.

Of all the preparations, I have never yet found any so effectual as calomel, and when 15 or 20 grains of arsenic are mixed with half a drachm of calomel, and given night and morning, I have never known them fail. Of the mineral kingdom, pewter, tin, and iron, are sometimes used. Tin is an efficacious anthelmintic for dogs.

### ASAFŒTIDA,

Is by some veterinarians considered as a minor antispasmodic; but having witnessed its good effects in giving it to the horse by the farriers of Asia, I think it worthy of a place in this pharmacopœia.

It is a gummy resinous substance, possessing a most powerful unpleasant smell. Among the Persians and natives of India, it is used as common in their cooking as garlic among the Spaniards, and as a human medicine among them is considered a good antispasmodic. Its use as a veterinary medicine is in the form of the fetid spirits of ammonia, and the dose is from one to five ounces. I have found it serviceable in coughs, and when employed as an expectorant squill is a useful addition.

### ASTRINGENTS.

The principal of the astringent medicines are the Gallic acid and Tannin; but those that are generally used for astringents, and those that act by constringing the divided ends of blood vessels, are called styptics; such as *chalk, alum, starch, opium,* and *catechu,* which act favourably in restraining internal fluxes, as diarrhœas, scouring, &c. Some chemists are, however, divided in their opinion respecting the acid and tannin, and some medical writers class the preparations of zinc, lead, iron, copper, &c. as tonics for debility.

### No. I.—*Astringents for Diarrhœa.*

| | |
|---|---|
| Prepared chalk . . . . | 4 drachms |
| Ginger . . . . . . | 2 drachms |
| Catechu . . . . . | ½ drachm |
| Opium . . . . . . | 1 drachm |

Mix into a ball with honey.

### No. II.

| | |
|---|---|
| Castile soap . . . . . | 1 drachm |
| Aromatic powder . . . . | 2 drachms |
| Alum . . . . . . . | 1 drachm |

Gum or honey for a ball,

No. III.

| | |
|---|---|
| Ol. Mint . . . . | 30 drops |
| Chalk . . . . . . | 5 drachms |
| Catechu . . . . | 1½ drachm |
| Gum and honey Q. S. for a ball | |

Note.—In cases of diarrhœa, when violent, I have found it good practice not to stop it too hastily; but to give first a gentle laxative before the astringent is administered.

No. I.—*Astringents for Diabetes.*

| | |
|---|---|
| Powdered ginger and oak bark, each . | 2 drachms |
| Opium . . . . . . . | 1 drachm |
| Which may be given in a pint of astringent decoction. | |

No. II.

| | |
|---|---|
| Alum . . . . . . | 1 ounce |
| Armenian bole . . . . . | 1 ounce |
| White vitriol . . . | 2 ounces |
| Rhubarb and honey, Q. S. for ball. | |

### ANTIMONY,

*(Antimonii Sulphuretum.)*

In veterinary medicine, is administered under various forms. It has long been used as an alterative, in doses from 5 to 10 drachms, and antimonial powder, or antimonium (*oxydum*) is a preparation from the crude, and simpler in its qualities than Dr. James's powder, which affords the practitioner an excellent febrifuge, in doses from a scruple to two drachms. Emetic Tartar (*Antimonium Tartarizatum*) is another and still more valuable medicine in horse practice. In all inflammatory, but more particularly in pneumonic catarrhal ones, it is perhaps one of the best remedies in the world, and may be given with safety to the horse in doses of from one to two drachms; and in gentler doses, mixed with about half a drachm of digitalis (*Fox Glove*), greatly tends to lower the action of the heart and arteries; though among farriers, the sulphuret or crude antimony levigated, is administered by them in very large doses.

NOTE.—The medicines enumerated in this pharmacopœia have undergone the test of fair experiment at the different veterinary institutions of Europe, under the inspection of some of the first veterinary medical practitioners perhaps in the world, and can therefore with safety be recommended as such.

J. CARVER,
*Professor of Animal Medicine.*

### VENTILATION.

Air for many ages has, by every considerate man, been considered of the greatest importance to the health and well-being of all vegetable and animal creation, and of late years, but more particularly among our present philosophers and chemists, has the labours of the veterinarian not been exerted in vain. Indeed of so much importance is it now considered, that the health of horses depends upon the salubrity of this grand and living principle, that with a view of obviating the many evils attendant on the want of it, and the little attention that was until lately paid to the ventilation of stables, that government have thought it one of the most salutary measures they could possibly adopt for the preservation and well-being of their cavalry. Professor Coleman has, by his experiments at the London Veterinary College, satisfactorily proved, that that fatal disease called the Glanders, can be generated in a few hours, by confining a horse in an impure air. Is it not notorious, that it is a common practice among grooms, but particularly those who *fancy themselves profoundly skilled in the art of farriery,* to stop every crevice they can find in the stable, so that pure air is with the greatest difficulty admitted, and the noxious vapours arising from the litter, from perspiration and respiration, are in a great measure confined. Let any person only take the trouble to walk through any of the public stables of this or any other city, during the intensely hot weather, and see what those animals must necessarily suffer from being penned up in the manner they are; and though the air may not be contaminated so far as to occasion immediate disease, no one will deny that it is not sufficient to debilitate the constitution, and thereby lay the foundation of numerous and sometimes fatal complaints, as well as create all those local diseases with which we daily and hourly see so many horses in all the livery stables suffering under; such as inflamed eyes, moon blindness, obstinate coughs, inflamed lungs, &c. injuries of the feet, &c.; all of which most certainly renders every animal more or less susceptible of cold than if he was exposed on a common; for I will maintain, that the more we follow the custom of placing a horse in a state of nature, under all circumstances of either sickness or health, the better will his health be preserved; and I will further maintain, that even in the depth of winter, a horse well clothed, and kept only in an open shed, will be less liable to contract disease, than a horse pampered in a fine stable, and often covered with heavy woollen clothing; and it is a fact that can be clearly proved, that a horse exposed even in the middle of winter on a common, is warmer, and feels more comfortable than one penned up in a fine stable in the city.

Mr. Coleman has therefore deserved well of his country, for introducing those salutary measures—the effect of ventilation. How much have I not pleaded?—how much have I not preached and argued their cause?—But all in vain. The only *satisfaction* that I have hitherto observed to result from those measures, is that of having gained myself a host of enemies, particularly among the lower order of men, who foolishly think their interests stabbed by my pointing out those things which might so easily be remedied. However, actuated by the best of motives, I still hope my reward is not very far distant, and whether those who think themselves so grievously injured by my preaching, like it or not, I feel a confidence that they must, in spite of all they can say or do, finally fall into this grand and salutary measure.

Heat, it is well known, is the grand object in nature both in the production of life and the communication of disease, according to the degree and manner in which it is applied. This being the case, the regulation and temperature of our stables in which the poor animal is to be kept, ought to be the first consideration with every owner, and of this he should be the judge himself, and not leave it to Tom, Dick or Harry. Say, if therefore the temperature of the external air be at 32 degrees, or the freezing point, surely it is preposterous that the stable should be at 60; the one being summer heat, and the other winter cold; and the moment the poor animal is brought out from the temperature of his stable, a thousand to one but he is in the course of a few hours attacked with inflamed lungs or some other inflammatory disease, from this very sudden change: and how easily all this might, I repeat it again and again, without expense, be prevented, and the poor animal saved from falling the martyr of disease.

It is not my design in this place to enter into a regular treatise on air, and the grand necessity of ventilation in all our public and private stables; but so much have I laboured to convince people of the propriety of preventing disease in this way, and the determined obstinacy that seems to prevail on almost all occasions, that I cannot but feel it my duty in noticing this subject on every opportunity that may occur, until I bring home conviction to their minds; and if any one should doubt the facts here stated, let him only walk through one of the public stables in the morning, and feel the extreme pungency of the heat and confined air, arising from the ammonia or volatile alkali which has disengaged itself during the night, and which by that time has become unfit for respiration—consequently destructive to the principles of life; then let him say whether I am correct in my observations or not. Let me now ask, if this extreme heat, which the poor animal has been suffering all night, does not *enervate and weaken* him, and render him less capable of going through the labours of the succeeding day? and which, from the relaxed state of the skin, must keep the horse constantly bathed in a copious perspiration.

If people would only consider the matter right, a stable to be really healthy should never be more than a few degrees warmer than the external atmosphere.—But then they cry out

"O John, my horse has got a rough coat!"—Let them consider the matter well, and ask themselves this question. Is it not better that my horse should have rather a rough coat and enjoy good health, than that I should cover him with woollen clothing, have every crack and crevice of the stable stopped to shut out his very best friend, and thereby make him liable to take on disease at every change of weather that may happen? The air for this purpose should always be admitted from below, and the aperture for its admission should be of sufficient size, and made so as to open and shut according to circumstances; but it should never be closed except in very intense cold weather.

The action of bad air is particularly apt to affect the eye of the horse, and a proof of it may be drawn from the well known fact, that diseased eyes chiefly occur among horses confined in warm stables. Though costiveness may aggravate the disease, we are convinced it cannot induce it; but the action of volatile alkali, both on the nose and eyes, is so notorious, that no shadow of doubt can remain, that if excessive heat once induces inflammation, this additional circumstance will generally increase it. Independent then of any other evil than inducing affections of the eye, the atmosphere of the stable should be kept perfectly cool and free, and its temperature only a little more than the external air. On the same account, as we have before observed, the size of stables should never be large, so as to crowd too many horses together. By this the air of the stable becomes vitiated, and contagion and disease might follow as the consequences.

